# Treating transgenic Alzheimer mice with a β-secretase inhibitor, what have we learned?

**DOI:** 10.18632/aging.100267

**Published:** 2011-01-17

**Authors:** Jordan Tang, Arun Ghosh

**Affiliations:** ^1^Protein Studies Program, Oklahoma Medical Research Foundation, University of Oklahoma Health Science Center, Oklahoma City, Oklahoma, USA; ^2^Departments of Chemistry and Medicinal Chemistry, Purdue University, West Lafayette, Indiana, USA

β-Secretase is an attractive target of amyloid-reduction therapy for Alzheimer's disease. Currently, no efficacy data is available from clinical trials of β-secretase inhibitors. Treating young transgenic Tg2576 mice with a brain-penetrating β-secretase inhibitor reduced brain amyloid-β by about 50% and rescued the age-related cognitive decline. Implications from these model studies on the design of clinical trials are discussed herein.

Since the identification of β-secretase (memapsin 2, BACE1) more than a decade ago, significant research effort from many laboratories around the world has been directed towards the development of inhibitor drugs against this β-secretase as a potential treatment of Alzheimer's disease (AD) [1]. The initial excitement and optimism were understandable. Since amyloid-β (Aβ) is an important driving force in AD pathogenesis, clinical intervention to reduce its production would be a logical therapeutic approach. Toward this end, the inhibition of β- or γ-secretase are the best choices for drug development, as these two proteases represent the first and second steps in the processing of amyloid precursor protein leading to Aβ production.

Inhibitor drugs for γ-secretase have exhibited toxicity problems in several clinical trials which appear to be rooted in the many important physiological functions of this protease, and possibly in the lack of alternative pathways to process its natural substrates when γ-secretase is inhibited. These are fundamental problems inherent to this target and have evaded practical solutions thus far. β-Secretase, on the other hand, is devoid of many of these problems. The inhibition of this first step in Aβ production may avoid other harmful late events in AD pathogenesis. Deletion of the β-secretase gene to eliminate Aβ production resulted in only minor phenotype changes in mice, suggesting that a partial reduction of Aβ generation by β-secretase inhibitors could be safe in clinical treatments. In addition, as an aspartic protease, β-secretase enjoys a successful precedence in the development of HIV protease inhibitor drugs used today.

The attractiveness of β-secretase as a drug target, however, has not translated into rapid pharmaceutical development. A decade after the target discovery, only a few drug candidates have entered the early phases of clinical trials and no successful demonstration of efficacy has been reported in human trials. The slow pace of pharmaceutical development coupled with the suspension of a number of β-secretase inhibitor projects in major companies, have earned β-secretase a perhaps deserving reputation of being a difficult target. In other words, it has been difficult to develop β-secretase inhibitors that possess all required properties such as blood-brain barrier penetration, selectivity, pharmaco-kinetic characteristics and toxicity. To a large extent, these difficulties are unique to this target. β-Secretase has a large active-site area for inhibitor binding. Yet it permits relatively limited options for designing small, potent and selective inhibitors and thus renders ineffective some of the traditional drug discovery tools, such as automated screening of chemical libraries or computer assisted inhibitor design.

However, by using crystal structure based inhibitor design, more drug-like inhibitors have emerged. At least three pharmaceutical companies have conducted Phase I trials for their inhibitor candidates; but convincing efficacy results from clinical trials will take years of future efforts. It is therefore appropriate to ask, what we have learned from therapeutic model, such as a transgenic AD mouse with β-secretase inhibitors? Although AD mice do not accurately duplicate human AD, they do share some early human AD features, which include excess production of brain Aβ, a decline of cognitive performance with age, and the development of amyloid plaques in the brain at middle-late age. AD mice do not progress to dementia as in human AD. However, for testing an amyloid reduction therapy using β-secretase inhibitors, the AD syndromes in these mice can produce useful insights to human treatments. At this stage in β-secretase inhibitor development, it would be informative to learn if these AD-like syndromes can be rescued in AD mice by partial amyloid reduction.

Chang *et al.* reported recently [2] that the administration of β-secretase inhibitor GRL-8234 rescued age-related cognitive decline and reduced brain amyloid burden in transgenic AD mice Tg2576. In this mouse strain, excess Aβ produced in the brain leads to cognitive decline starting at about 6 months of age and continues through life. At about 10 months of age, amyloid plaques start to appear in the brain. The age-related cognitive decline in this strain of AD mice [3], which is dependent on the overproduction of Aβ in the brain, is the most stringent efficacy criteria available in AD mouse model. In the studies of Chang *et al.*, AD mice were continuously infused with GRL-8234 which reduced brain and plasma Aβ by about 50% of the controls. In three separate experiments in which the starting age of the mice ranged from 5.5 to 9 months, differences in the cognitive performance between the treated and the control mice were seen only after a period ranging from about 4 to 7 months. Treatments of shorter periods or starting treatment at an older age (18 months) failed to show the benefit of cognitive rescue. In all the experiments which resulted in cognitive rescue, the reduction of brain amyloid was about 50%.

These results are encouraging and several interesting points have emerged from this study. First, a partial reduction of Aβ production and brain plaque load of about 50% was sufficient to rescue the cognitive decline in this model. The current results appear to suggest that the cognitive decline can be significantly improved in spite of some excess of brain Aβ. Conceivable mechanisms to accommodate these findings may include the presence of an Aβ threshold for cognitive decline or partial Aβ reduction may delay the onset of cognitive decline. Regardless of the mechanism, efficacy of cognitive rescue by partial Aβ reduction would be good news if it is confirmed by clinical studies. Second, there was no overt toxicity associated with treatment over a 7 month time period and no significant accumulation of APP in the brain of treated mice, a potential mechanism based side effect. The latter observation suggests that the known alternative APP processing pathway by α-secretase was sufficiently active to dispose an overproduced β-secretase substrate. Third, it took several months, which was somewhat surprising to manifest cognitive rescue in inhibitor treated mice. One explanation is that the cognitive decline in Tg2576 is known to be a slow process. Thus, the differential of cognitive performance between the treated and control groups would require a long time. Fourth, inhibitor treatment of old mice about 6 months after the appearance of brain plaque failed to show the benefit. This failure may stem from the fact that the old mice became incompetent for cognitive tests before the rescue could be demonstrated. Another possibility is that some of the amyloid existed in the brain of old mice prior to the beginning of the treatment may serve as a source of Aβ in a partial resolubilization process. This may have kept Aβ levels higher than expected and thus would require an even longer time to show cognitive rescue. It is perhaps worthwhile to consider the last two points in the setting of human clinical trials. In AD patients, cognitive performance declines slowly in general and amyloid plaques are present in the later stages of the disease. For clinical trials of β-secretase inhibitor drugs, cognitive tests are the only efficacy criteria acceptable for the FDA. Therefore, a Phase II/III trial with early stage AD patients over a long trial period will have the best chance to avoid the potential problems discussed above. In addition, testing β-secretase inhibitor drugs in an amyloid reduction concept, early stage AD patients would have less brain destruction and inflammation, which is not expected to be reversed quickly by β-secretase inhibitors and may obscure the benefit of the drugs. It will be of interest to study the same β-secretase inhibitor in both AD mice and human efficacy trials. A predictive model may contribute to the planning and decisions on long, costly human trials.
